# Unusual tumours of the heart: diagnostic and prognostic implications

**DOI:** 10.1186/1749-8090-4-4

**Published:** 2009-01-12

**Authors:** Zeena Makhija, Ranjit Deshpande, Jatin Desai

**Affiliations:** 1 Kings College Hospital, London, UK

## Abstract

Metastases to the heart are extremely uncommon. We describe three unusual cases along with their management. A review of the current literature concerning cardiac secondaries is included.

## Background

Cardiac tumours are a reclusive subdivision of oncology. Though they are classified as either primary (benign or malignant) or secondary, we still lack a definitive classification based on aetiology, histopathology or a system of staging for the tumours of the heart. Primary tumours of the heart are mostly myxomas whereas as secondary deposits preferentially come from melanoma, primary mediastinal tumours, breast and colon [1].

Rectal cancer is the second most common cause of death in the United Kingdom (UK). Over 30,000 new cases are diagnosed every year. Despite the advent of new oncological strategies, 8–15% of patients develop distant metastases within the first five years after initial presentation [2]. In women worldwide, breast cancer is the second most common cause of death. Eight percent patients present with distant metastases. The commonest sites of metastatic spread of rectal and breast cancer are bone, pleura, liver, peritoneal cavity, lung and the brain [3]. In the UK, the incidence of oral cancer is low at 1%, as compared to South East Asia. The 5 year survival rate of oral cancer is as low at 50% due to late presentation and increased risk of developing a second malignancy. Secondly local recurrence is more common than distant metastases [4].

We describe three cases of metastatic rectal, breast and tongue carcinoma with secondaries to the heart mimicking thrombus or myxoma.

## Case presentations

### Case 1

A 70-year old gentleman was admitted with NYHA grade III dyspnoea. His co-morbidities included type II diabetes mellitus, hypertension and recent onset atrial fibrillation (AF). He was being treated for rectal carcinoma (T3N2M1) with chemotherapy.

**Figure 1:**
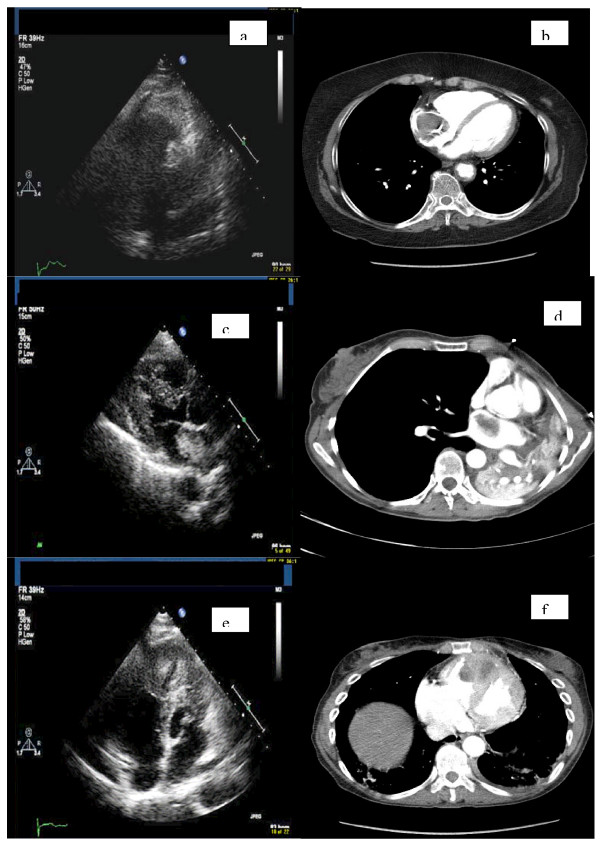
**a. Echocardiographic image of case 1 showing the tumor in right atrium**. b. CT image of case 1 showing the tumor. c. Echocardiographic image of case 2 showing the tumor in left atrium. d. CT image of case 1 showing the tumor. e. Echocardiographic image of case 3 showing the tumor in right ventricle. f. CT image of case 3 showing the tumor.

CT scan on admission showed no change in pulmonary metastases (Figure 1b). Trans-thoracic echocardiography (TTE) revealed a mass in the right atrium (Figure 1a). Provisional diagnoses of thrombus, myxoma and metastasis were considered.

A limited wide excision was performed via a median sternotomy. There was a large, firm, lobulated mass invading the entire right atrial wall extending to the tricuspid valve. The right atrium was reconstructed using allogenous pericardial patch.

Histopathology report indicated it was metastatic rectal adenocarcinoma. Inspite of further chemotherapy, CT scan at 6 weeks showed advanced pulmonary metastases and residual mass in the right atrium.

### Case 2

A 59-year old lady was investigated for sudden onset NYHA grade III dyspnoea and recent onset AF. She had undergone radical left mastectomy for invasive ductal carcinoma, 11 years earlier. She currently had liver and bone metastases.

CT scan revealed a mass in the left main bronchus with a filling defect in the left atrium (Figure 1d). TTE confirmed a mobile, heterogeneous mass in left atrium suspected to be a myxoma (Figure 1c).

Multiple biopsies were taken at bronchoscopy. Silver methenamine stain was definitive for Aspergilloma. She received 4 weeks of antifungal therapy. A TTE at 8 weeks revealed that the mass had decreased in size from 7.4 cm^2 ^to 6.5 cm^2^. A trans-oesophageal biopsy revealed features consistent with myxoma. In view of her advanced malignancy and limited life expectancy, it was agreed that the surgical removal of the mass would entail major resection using cardiopulmonary bypass with an unfavourable risk-benefit ratio. A CT scan 3 months later confirmed extensive spread to the lung parenchyma and the mediastinum, with no further decrease in the size of cardiac mass. She is currently receiving palliative care.

### Case 3

A 66-year old lady presented with classical anginal symptoms in the casualty. 3 years ago, she had undergone radical resection for tongue carcinoma.

Her ECG on presentation showed 1.5 mm ST elevation in lead V1 and 2.5 mm in lead V2. She was treated for acute myocardial infarction.

Coronary angiogram revealed 50% stenosis of the left anterior descending artery and the first obtuse marginal branch of the left circumflex artery. TTE revealed a large right ventricular (RV) mass obstructing the right ventricular outflow tract (Figure 1e). CT confirmed the presence of RV mass along with a left hilar mass compressing the left upper lobe bronchus and the pulmonary veins (Figure 1f). Bronchoscopic biopsies revealed it was metastatic Squamous Cell Carcinoma (SCC) from the tongue. A trans-oesophageal biopsy revealed features consistent with tongue SCC. She was referred for palliative therapy in view of the advanced nature of the disease.

## Discussion

Although secondary cardiac tumours are rare, their reported incidence is between 1.6% and 20.6% [5]. The most common sources of cardiac metastases include lung, breast and the lymphoreticular malignancies [6]. Though sporadic cases of metastases from colonic cancer to the heart have been reported, there has been no report so far of invasion of the endocardium from a rectal primary [7]. Histopathologically, the commonest tumour to metastasize to the heart has been adenocarcinoma and the preferential sites have been the pericardium and the epicardium. The regions of the heart affected, in increasing order of frequency, are the endocardium, myocardium, and pericardium.

Non-cardiac tumours may disseminate to the heart by lymphatic or haematogenous pathways, local spread, or a transvenous extension. Retrograde spread through the lymphatic channels originating in the mediastinum and reaching the heart is the most common route of extension [8]. Haematogenous spread can occur via the coronary circulation or rarely, through the vena cavae. Since most of the cancerous cells are filtered by the hepatic and pulmonary microcirculation, haematogenous metastases to the heart are associated with metastases to these organs as well.

Transvenous extension of the tumour thrombus can occur to the chambers of the heart via superior/inferior vena cavae (to the right atrium) or the pulmonary veins (to the left atrium). The former is most commonly seen in patients with renal cell carcinoma [9].

The dyspnoeic symptoms of patient in case 1 were neither acute or subacute in onset and disproportionate to his radiographic findings. i.e. the pleural effusions on CT scan were causing blunting of bilateral costophrenic angles and had neither increased or decreased over the last three months. Since the pulmonary metastases were unchanged and in the presence of atrial fibrillation with bilateral pleural effusions cardiac failure appeared to be the likely aetiology. Although there was rectal malignancy no CT lesions suggestive of cardiac metastases were seen. Histology revealed multilayering of glandular epithelium and extra cellular mucin production suggestive of poorly differentiated adenocarcinoma. On retrospective review of the rectal biopsy specimen its histological appearances were found to be similar. Hence the cardiac tumour was found to be consistent with metastatic carcinoma of the rectum.

If we hypothesise the tumour spread route, direct invasion and lymphoreticular spread are ruled out because of obvious anatomical reasons. If the tumour emboli, seeded through the portal circulation, via liver and the inferior vena cava and lodged into the right atrium before seeding into the lungs, it brings to light two reasonable assumptions. Firstly, it is possible for the rectal primary to invade the heart without seeding into the liver and secondly, patient's dyspnoea and atrial fibrillation were secondary to the involvement of the heart before that of the lungs. Sudden onset of arrhythmias is indicative of metastatic spread to the myocardium. These could be the result of direct invasion of the autonomic fibres, coronary arteries or indirectly caused by factors such as hypoxemia, anaemia or electrolyte imbalances [9,10].

A review of the intra-operative findings reveals that the tumour was debulked in view of its aggressive and invasive nature. This compared with the lesions in the lung, which have remained stable, could be attributed to the fact that the tumour micro emboli were filtered out in the heart before reaching the lung.

In the second case, the histology revealed the left atrial mass was a myxoma. She developed opportunistic aspergillus infection being immunocompromised secondary to chemo-radiation. Aspergillus fumigatus is a dimorphic fungus. It is the most frequent opportunistic infection followed by Candida, Nocardia and Mucormycosis in immunocompromised patients [11]. Lung is the commonest site of infection. Since this patient was severely immunocompromised secondary to chemo-radiation she developed pulmonary infection. Silver methenamine stain was definitive for Aspergillus culture of sputum and bronchial washings. This finding of aspergilloma was incidental secondary to low suspicion of pulmonary metastases. There are few case reports of the co-existence of aspergilloma with lung cancer [12]. Aspergillus can also involve blood vessels and the central nervous system. The left atrial mass found in this case was thought to be an Aspergillus ball due to the reduction in size on serial TTE, good clinical improvement and clinical response to anti-fungal therapy. Hence she was treated medically. However within three months she deteriorated with extensive mediastinal spread of pulmonary metastases. In retrospect if the Aspergillus would have propagated via the systemic circulation to the heart the patient would have been in septic and cardiogenic shock. Secondly on considering the invasiveness of her breast cancer, in spite of the itraconazole therapy the bronchial mass continued to spread throughout the lung and the mediastinum. The presence of a mobile intra-cardiac mass in a treated case of breast cancer should lead to suspicion of recurrence. The relevance of including this case in our reported series is to highlight the co-existence of a primary cardiac tumor with aspergillosis which usually co-exists with maligancies with secondary spread that lead to an immunocompromised state. Myxoma with superimposed aspergillus co-infection has not been reported so far.

In the last case, squamous cell carcinoma was the predominant histological type of tongue cancer. Smokers with head and neck cancers are at an increased risk of developing a second primary within two years of the first one [13]. This is proposed by the field cancerisation theory based on the extensive field effect of aetiological agents such as tobacco and nicotine. Oral cancers are known for skip metastases without loco regional occurrence [14]. Angioinvasion is deemed to be the modality of spread. In this lady, lesions in the lung, mediastinum and the heart were detected three years after complete response of the primary. She had no cervical lymphadenopathy. The presence of secondaries in the lungs and heart in the absence of cervical spread, suggests that haematogenous route was adopted by the primary. Historically these metastatic lesions were uncommon, however the advances in early diagnosis, coupled with radical surgery or chemoradiotherpy regimen for oral cancer has lead to longer survival rates and may have increased the late detection of skip lesions.

Scanning for metastases to the heart is not a routine practice for patients with malignancy. This report addresses the fact that there is a lack of screening protocol for patients with cardiac metastases. Currently, Uncertainties exist in deciding appropriate investigation modalities and medical/surgical management of such patients. A multicenter registry may provide more data to decide the best diagnostic approach and establish the role of surgical excision for palliation in this subgroup.

From our experience scanning the heart for possible metastases may be indicated in cases with pulmonary or hepatic spread and presence of atrial arrhythmias. Since the TTE is sensitive and specific for detecting intracardiac tumours it might be the investigation of choice in these patients. The value of trans-oesphageal biopsy and prognostic value of screening needs more conclusive evidence.

## Consent

Written informed consent was obtained from the patients for publication of this case report and any accompanying images. A copy of the written consent is available for review by the Editor-in-Chief of this journal.

## Competing interests

The authors declare that they have no competing interests.

## Authors' contributions

ZM collected the data on all three cases and has written the article. RD was the consultant cardiothoracic surgeon to whom cases 2 and cases 3 were referred. JD was the consultant cardiothoracic surgeon who operated on case 1. He is also the senior author for this case report who has supervised all revisions and the original paper.
